# Extracellular serglycin upregulates the CD44 receptor in an autocrine manner to maintain self-renewal in nasopharyngeal carcinoma cells by reciprocally activating the MAPK/*β*-catenin axis

**DOI:** 10.1038/cddis.2016.287

**Published:** 2016-11-03

**Authors:** Qiaoqiao Chu, Hongbing Huang, Tiejun Huang, Li Cao, Lixia Peng, Simei Shi, Lisheng Zheng, Liang Xu, Shijun Zhang, Jialing Huang, Xinjian Li, Chaonan Qian, Bijun Huang

**Affiliations:** 1 Department of Pharmacy, Sun Yat-Sen University Cancer Center, Guangzhou, China; 2 Department of Nuclear Medicine, The Second People's Hospital of Shenzhen, Shenzhen, China; 3 Department of Experimental Research, State Key Laboratory of Oncology in South China, Collaborative Innovation Center for Cancer Medicine, Sun Yat-Sen University Cancer Center, Guangzhou, China; 4 Department of Medical Oncology, Sun Yat-Sen University Cancer Center, Guangzhou, China; 5 Department of Traditional Chinese Medicine, The First Affiliated Hospital of Sun Yat-Sen University, Guangzhou, China; 6 Department of Pathology, Saint Barnabas Medical Center, Livingston, NJ, USA; 7 Department of Neuro-Oncology, The University of Texas MD Anderson Cancer Center, Houston, TX, USA; 8 Department of Nasopharyngeal Carcinoma, Sun Yat-Sen University Cancer Center, Guangzhou, China

## Abstract

Serglycin is a proteoglycan that was first found to be secreted by hematopoietic cells. As an extracellular matrix (ECM) component, serglycin promotes nasopharyngeal carcinoma (NPC) metastasis and serves as an independent, unfavorable NPC prognostic indicator. The detailed mechanism underlying the roles of serglycin in cancer progression remains to be clarified. Here, we report that serglycin knockdown in NPC cells inhibited cell sphere formation and tumor seeding abilities. Serglycin downregulation enhanced high-metastasis NPC cell sensitivity to chemotherapy. It has been reported that serglycin is a novel ligand for the stem cell marker CD44. Interestingly, we found a positive correlation between serglycin expression and CD44 in nasopharyngeal tissues and NPC cell lines. Further study revealed that CD44 was an ERK-dependent downstream effector of serglycin signaling, and serglycin activated the MAPK/*β*-catenin axis to induce CD44 receptor expression in a positive feedback loop. Taken together, our novel findings suggest that ECM serglycin upregulated CD44 receptor expression to maintain NPC stemness by interacting with CD44 and activating the MAPK/*β*-catenin pathway, resulting in NPC cell chemoresistance. These findings suggest that the intervention of serglycin/CD44 axis and downstream signaling pathway is a rational strategy for targeting NPC cancer stem cell therapy.

Nasopharyngeal carcinoma (NPC) is one of the most common malignancies in southern China and Southeast Asia.^1, 2^ The standard treatment for early-stage NPC is radiotherapy, and the treatment for advanced NPC is concurrent chemoradiotherapy.^3, 4, 5^ However, approximately 30% of patients have significant rates of local relapse or distant metastasis, and the prognosis is very poor for these patients.^6^ Metastatic NPC cells often develop chemotherapy resistance, but the molecular mechanisms underlying NPC metastasis and chemotherapy resistance are not fully understood.

Serglycin is a proteoglycan expressed in hematopoietic cells, endothelial cells and embryonic stem cells,^7, 8, 9^ and it contains a 17.6 kDa core protein to which eight heparin or chondroitin sulfate glycoaminoglycan chains are attached. The core protein is rich in serine–glycine repeats. The glycoaminoglycan chains vary depending on cell type and have an important impact on serglycin function.^10^ Although serglycin does not contain a transmembrane domain, it can be constitutively secreted by hematopoietic cells, endothelial cells, pancreatic acinar cells and myeloma cells.^9, 11, 12, 13^

As an extracellular matrix (ECM) component, serglycin participates in packaging secretory granules and regulates the storage or release of enzymes, serotonin and histamine in hematopoietic cells.^14, 15^ We first reported that serglycin promoted NPC epithelial-to-mesenchymal transition (EMT) and metastasis in an autocrine manner.^16^ Recent results showed serglycin promoted the aggressive phenotype of breast cancer cells metastasis and was associated with tumorigenesis in myeloma and acute myeloid leukemia.^12, 17, 18, 19, 20^

In our previous study, we performed genomic expression profiling of high-metastic and low-metastic NPC cell lines and corresponding xenograft tumors and found that serglycin was the second most highly upregulated gene and associated closely with metastatic NPC cells *in vitro* and *in vivo*.^16^ Subsequently, we further showed that serglycin was an independent marker of distant metastases in NPC.^21^ In another study comparing the genomic expression profiles of NPCs *versus* non-cancerous naspharyngeal tissues, we found that the ECM remodeling pathway was the most significantly changed signaling pathway in NPC tissues.^20^ Our findings suggest that serglycin proteoglycan acts as microenviroment ECM, where NPC cancer stem cells (CSCs) reside, and may have an important role in ECM remodeling responsible for NPC progression. Serglycin as a ligand recognizes CD44 receptor, which is a marker of CSCs.^18, 22, 23^ These results suggest that serglycin/CD44 axis have an important role in maintaining stem cell self-renewal. However, the signaling pathway by serglycin/CD44 axis activation is so far unknown in any hematological and epithelial malignances. In this study, we demonstrate that serglycin is closely associated with CSC properties. Serglycin serves as a novel CD44 ligand, which is a downstream target of *β*-catenin signaling. Our findings first revealed that serglycin could maintain NPC cell stemness by upregulating CD44 and activating the MAPK/*β*-catenin pathway to modulate NPC metastasis and chemotherapy resistance.

## Results

### Highly metastatic S18 cells have more significant cancer stem-like cell characteristics compared with lowly metastatic S26 cells

**Figure 1:**
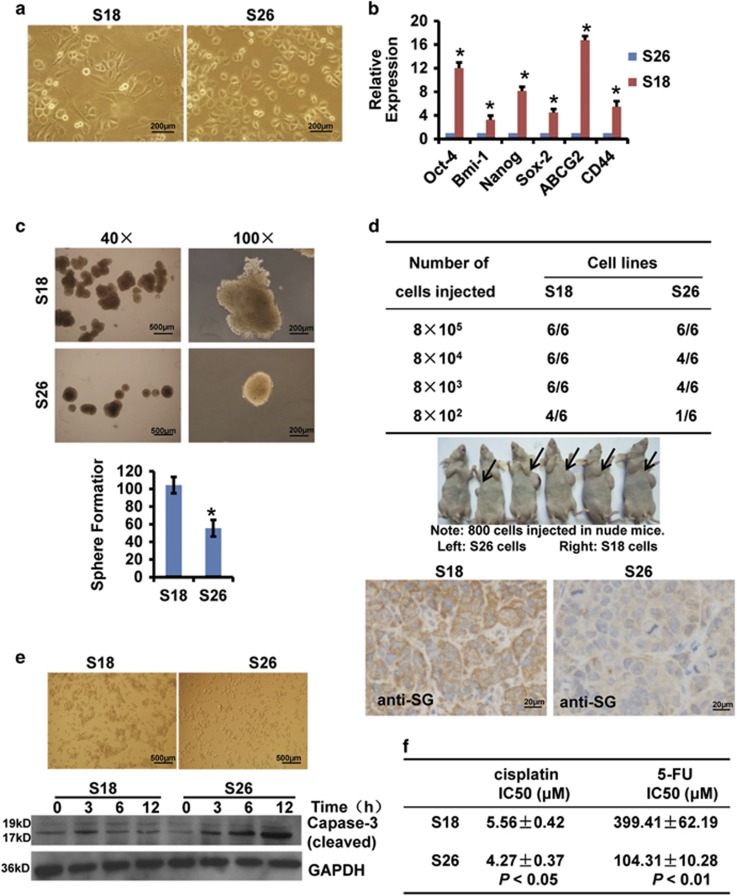
S18 cells have more significant cancer stem-like cell characteristics. (**a**) The differences in cellular morphology between S18 cells and S26 cells. (**b**) mRNA expression levels of stem cell-associated genes (normalized to GAPDH) in S18 and S26 cells. Data represent the average±S.D., *n*=3; *P<0.05. (**c**) Sphere formation assay. S18 and S26 cells were counted and plated in ultra-low cluster plates for 10 days to allow for tumor sphere formation (top panel). The number of spheres is shown in the bottom panel. Data represent the average±S.D., *n*=3; *P<0.05, Student's *t*-test. (**d**) Tumorigenic assay. Serial dilutions of S18 and S26 cells were subcutaneously injected into nude mice and were monitored twice per week for 30 days. IHC staining of serglycin in S18 tumor tissues and S26 tumor tissues (bottom panel, 200 ×) . (**e**) S18 and S26 cells were seeded into ultra-low cluster plates and photographed at × 40 magnification after 6 h (top panel). The expression of cleaved caspase-3 was determined by immunoblotting (bottom panel). (**f**) The IC_50_ of cisplatin and 5-fluorouracil in S18 and S26 cells after 48 h. Data represent the average±S.D., *n*=3

The highly metastatic S18 cells and lowly metastatic S26 cells are two single subclones isolated from the parental CNE2 NPC cell line by our lab.^24^ S26 cells were primarily cobblestone-shaped, whereas S18 cells exhibited a spindle-shaped EMT-like morphology (Figure 1a). Recent studies have shown that CSCs are highly metastatic and may be quite plastic and associated with EMT.^25, 26^ Therefore, we speculated that S18 cells would have more cancer stem-like cell characteristics. To confirm this hypothesis, we examined the expression of stem cell-associated genes, including Oct-4, Bmi-1, Nanog, Sox-2, ABCG2 and CD44. We found that S18 cells expressed higher levels of these genes compared with S26 cells (Figure 1b).

We next performed sphere formation assays to evaluate the self-renewing capacity of these two cell lines. S18 cells formed larger and more abundant tumor spheres than S26 cells (Figure 1c). More importantly, when 8 × 10^2^ S18 cells were injected subcutaneously into nude mice, 66.7% of the mice (4/6) developed tumors compared with only 16.7% of the mice (1/6) injected with S26 cells. These observations suggest that S18 cells have a stronger tumor seeding and initiating ability than S26 cells. Immunohistochemistry (IHC) showed that S18 tumor tissues highly expressed serglycin in the stroma (Figure 1d). Highly metastatic cells are more likely to avoid anoikis.^27^ To examine the cells' ability to resist anoikis, we transferred S18 cells and S26 cells from adhesive cell culture dishes to ultra-low cluster plates that inhibit cellular attachment. We found that S18 cells clumped together, whereas most S26 cells remained as single cells and expressed higher levels of cleaved caspase-3 (Figure 1e), suggesting that S18 cells avoid anoikis. Furthermore, considering that cisplatin and 5-fluorouracil are the first choice of chemotherapeutic drugs for NPC clinical patients, we determined the IC_50_ of cisplatin and 5-fluorouracil in each cell line and found that S18 cells were more resistant to chemotherapeutic drugs than S26 cells (Figure 1f). These data suggest that S18 cells have more cancer stem-like cell characteristics than S26 cells, implying that S18 is a desired cellular model for NPC CSC research *in vitro* and *in vivo*.

### Serglycin overexpression is closely associated with the CSC-like properties of S18

**Figure 2:**
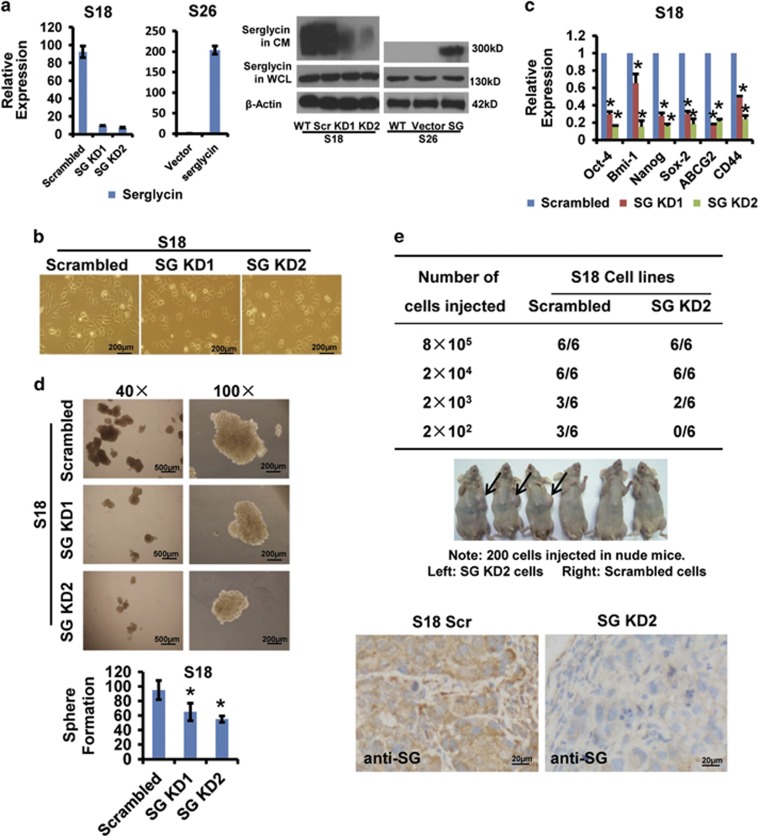
Serglycin suppression reduces CSC-like properties of S18 cells *in vitro* and *in vivo*. S18 cells were stably transfected with one of two serglycin shRNAs (KD1, KD2) or scrambled shRNA. (**a**) The expression of serglycin was detected by quantitative real-time PCR and immunoblotting in S18 and S26 stable cell lines. Serglycin protein levels were not changed in whole-cell lysates (WCL), but secreted serglycin protein was significantly reduced in CM after serglycin knockdown. (**b**) The change in cellular morphology of stably transfected cells (40 ×) . (**c**) Serglycin knockdown markedly decreased the expression of stem cell-associated genes. Data represent the average±S.D., *n*=3; *P<0.05 (**d**) Serglycin suppression reduced the size and number of spheres generated by S18 stable cells. Data represent the average±S.D., *n*=3; *P<0.05 relative to the scrambled controls. (**e**) S18 scrambled cells or S18 SG KD2 cells were subcutaneously inoculated into nude mice and were observed twice per week for 30 days. IHC staining of serglycin in S18 scrambled tumor tissues and in S18 SG KD2 tumor tissues (bottom panel, 200 ×)

Our previous studies showed that serglycin expression increased in highly metastatic S18 cells and corresponding xenograft tumors but not in lowly metastaic S26 cells.^16^ We next stably decreased serglycin mRNA and protein expression in S18 cells using two different lentiviral vector-carrying shRNAs (SG KD1 and SG KD2). Noticeably, we confirmed that secreted serglycin protein with approximately 300 kDa molecular weight was significantly decreased in conditioned medium (CM), whereas the cytoplasmic serglycin protein with 130 kDa molecular weight showed no different abundance via immunoblotting, which suggesting that the secreted serglycin ECM was more highly glycosylated and that serglycin functioned in an autocrine manner. On the other hand, in S26 cells overexpressing serglycin, secreted serglycin protein with approximately 300 kDa molecular weight was significantly increased in CM. (Figure 2a). Interestingly, the cellular morphology of S18 changed from spindle-shaped to cobblestone-shaped resembling S26, indicating that following stable knockdown of serglycin, the highly metastic S18 cells underwent mesenchymal-to-epithelial transition (MET) (Figure 2b). Serglycin knockdown decreased the expression of stemness-related genes, including Oct-4, Bmi-1, Nanog, Sox-2, ABCG2 and CD44 (Figure 2c). Serglycin suppression also reduced the size and number of spheres generated by S18 cells, indicating serglycin enhanced S18 self-renewal capacity (Figure 2d). In addition, when 2 × 10^2^ cells were injected into nude mice, 50% of the mice (3/6) formed palpable tumors in the S18 scrambled cells group compared with no mice (0/6) in the S18 SG KD2 group, suggesting that serglycin level was associated with tumorigenesis or tumor-initiating capacity *in vivo*. IHC results also showed decreased serglycin expression in S18 SG KD2 tumor tissues (Figure 2e). However, serglycin knockdown did not affect the cellular growth rate when cells were cultured in 10% fetal bovine serum (FBS). However, serglycin knockdown cells had a slower growth rate compared with S18 scramble control cells when the cells were maintained in medium containing 0.1% FBS, which suggesting that ECM serglycin promoted CSC-like S18 cells survival in serum-depleted microenvironment (Supplementary Figure S1A). These results strongly suggest that serglycin is closely associated with the CSC-like properties of S18 cells.

### Serglycin downregulation enhances S18 cell chemotherapy sensitivity

**Figure 3:**
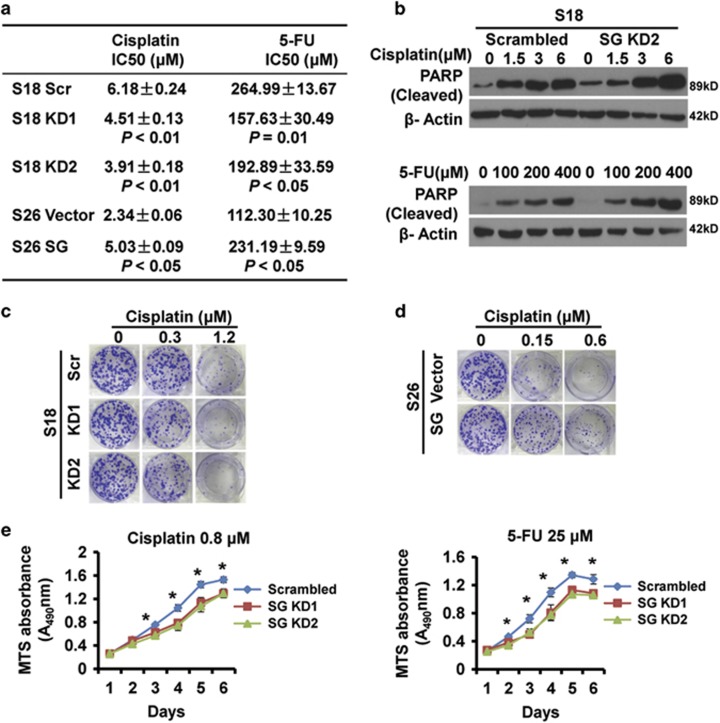
Serglycin knockdown enhances cisplatin and 5-fluorouracil sensitivity of S18 cells. (**a**) Serglycin knockdown S18 (KD1 and KD2) cells, scrambled control cells, serglycin overexpress S26 cells and vector cells were treated with various concentrations of cisplatin or 5-fluorouracil for 48 h to determine the IC_50_ by MTS assays. (**b**) S18 scrambled cells or S18 SG KD2 cells were treated with increasing doses of cisplatin or 5-fluorouracil for 48 h, and cleaved PARP expression was analyzed by immunoblotting. (**c**) Colony formation assays of stably transfected S18 cells treated with a range of cisplatin doses (0, 0.3, 1.2 *μ*M). (**d**) Colony formation assays of stably transfected S26 cells treated with a range of cisplatin doses (0, 0.15, 0.6 *μ*M). (**e**) Proliferation of stably transfected S18 cells treated with cisplatin (0.8 *μ*M) or 5-fluorouracil (25 *μ*M) for 6 days. Data represent the average±S.D., *n*=3; *P<0.05 for KD1 or KD2 cells compared with scrambled controls

CSCs are usually more resistant to chemotherapy-induced apoptosis in comparison with non-CSCs. S18 cells were resistant to chemotherapeutic drugs (Figure 1f), and it has been shown that serglycin was more highly expressed in drug-resistant hematopoietic cell lines.^28^ To further investigate the contribution of ECM serglycin to chemoresistance in S18 cells, we determined the IC_50_ of cisplatin and 5-fluorouracil and found that serglycin knockdown cells were more sensitive to cisplatin and 5-fluorouracil than the control cells. In S26 cells, serglycin overexpressed cells were resistant to cisplatin and 5-fluorouracil than the control cells (Figure 3a). Moreover, serglycin knockdown cells had higher cleaved PARP levels than control cells, confirming that serglycin confers resistance to cisplatin or 5-fluorouracil-induced apoptosis (Figure 3b). Colony formation assays revealed that serglycin knockdown reduced the number of cisplatin-treated (0.3, 1.2 *μ*M) S18 cell colonies (Figure 3c), and serglycin overexpression increased the number of cisplatin-treated (0.15, 0.6 *μ*M) S26 cell colonies (Figure 3d). In addition, serglycin knockdown inhibited cell growth rate upon cisplatin (0.8 *μ*M) or 5-fluorouracil (25 *μ*M) treatment, as examined by MTS assay (Figure 3e). These data highlight the important survival role of serglycin as niche ECM in the chemotherapy resistance of CSC-like S18 cells.

### NPC CSC surface marker CD44 is an ERK-dependent downstream serglycin effector

**Figure 4:**
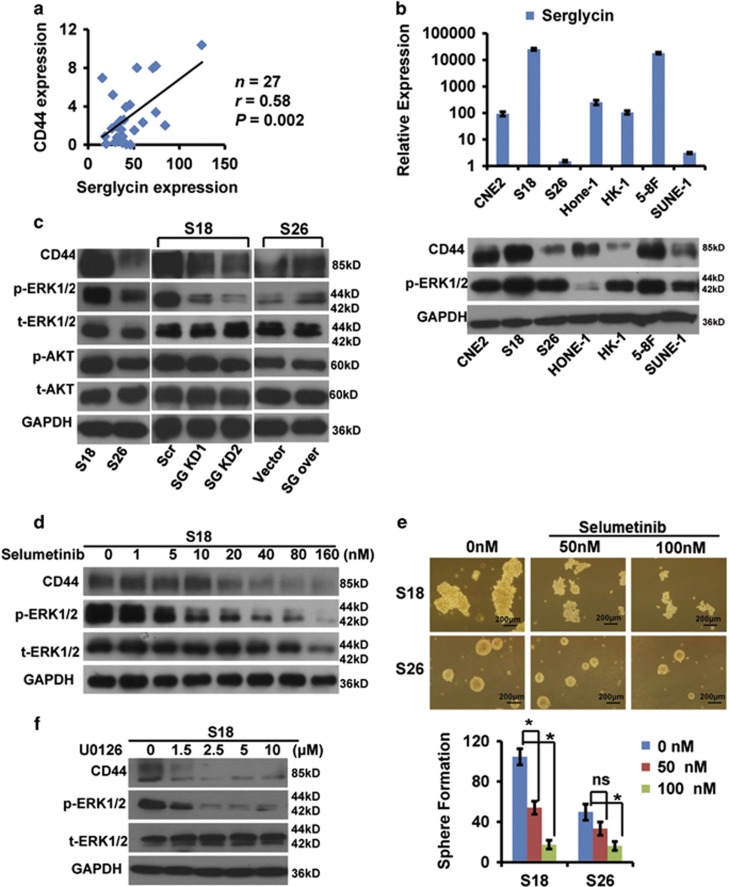
Highly correlated expression levels between serglycin and CD44. (**a**) Serglycin mRNA levels were positively correlated with CD44 expression in nasopharyngeal tissues as determined by quantitative real-time PCR (GAPDH was used as a reference gene. Pearson analysis, *r*=0.58; *P*=0.002). (**b**) Relative serglycin mRNA levels (normalized to GAPDH) in seven NPC cell lines as assessed by quantitative real-time PCR (top panel). Western blot analysis of CD44 expression in seven NPC cell lines. GAPDH was used as a loading control (bottom panel). (**c**) Western blot analysis of CD44, p-ERK1/2, t-ERK1/2, p-AKT and t-AKT in the indicated cell lines. SG over or vector represent S26 cells stably overexpressing serglycin or transfected with empty vector, respectively. (**d**) Western blot analysis of whole-cell lysates from S18 cells treated with increasing doses of selumetinib (ERK inhibitor) for 48 h. (**e**) The effect of selumetinib (0, 50, 100 nM) on tumor sphere formation by S18 or S26 cells. The number of spheres is shown in the bottom panel. Data represent the average±S.D., *n*=3; *P<0.05 *versus* 0 nM-treated cells. (**f**) Western blot analysis of whole-cell lysates from S18 cells treated with increasing doses of U0126 (ERK inhibitor) for 12 h

Although the CSCs surface marker are not well established in NPC cells by now, our recent investigations showed that CD44 was likely to be a desired surface CSC marker candidate in NPC cells.^29, 30, 31^ To confirm the relationship between ECM serglycin ligand and its receptor CD44, we examined their expression in 27 nasopharyngeal samples by using quantitative real-time PCR and found a strong positive correlation (*r*=0.58, *P*=0.002) (Figure 4a). Additional expression analysis of seven NPC cell lines demonstrated that serglycin levels (Figure 4b, top panel) correlated with higher CD44 levels (Figure 4b, bottom panel). Notably, we detected higher phospho-ERK1/2 levels in several NPC cell lines with higher serglycin and CD44 expression, including highly metastatic S18 and 5-8 F cell lines (Figure 4b, bottom panel). The above results suggested that ECM serglycin ligand trended to work in coordination with its receptor CD44, accompanied by activation of MAPK pathway.

To explore serglycin-induced signaling pathways, we first examined total and phosphorylated ERK, AKT and CD44 protein levels in S18 and S26 cell lines. We generated S26 cells stably overexpressing serglycin or transfected with empty vector and confirmed serglycin expression in these cells by quantitative real-time PCR and western blot analysis (Supplementary Figure S1B). Serglycin was overexpressed and secreted into the culture in S26 SG over cells compared with S26 vector cells without detectable increasing cytoplasmic protein by western blotting (Supplementary Figure S1B). S18 cells expressed significantly higher levels of CD44, phospho-ERK1/2 and phospho-AKT compared with S26 cells (Figure 4c, left panel). We subsequently determined the expression of the same proteins after serglycin knockdown or overexpression by western blot analysis. Serglycin knockdown S18 cells displayed decreased CD44 and phospho-ERK1/2 levels, whereas phospho-AKT levels did not change (Figure 4c, middle panel). In contrast, serglycin overexpression in S26 cells increased CD44 and phospho-ERK1/2 expression but had no effect on phospho-AKT levels (Figure 4c, right panel). The results above indicated that ECM serglycin-mediated modulation of its receptor CD44 was in an ERK-dependent and AKT-independent way.

The specific ERK inhibitor selumetinib effectively suppressed phospho-ERK1/2 expression in S18 cells and profoundly inhibited NPC CSC marker CD44 expression in a dose-dependent manner (Figure 4d). Interestingly, 50 nM selumetinib treatment did not significantly inhibited S26 cell sphere formation, but markedly decreased the number of S18 cell spheres (Figure 4e). Moreover, in S18 cells, another ERK inhibitor U0126 also inhibited CD44 expression and cell sphere formation in a dose-dependent manner (Figure 4f,Supplementary Figure S1B). Taken together, these findings indicate that NPC CSC marker CD44 is an ERK-dependent downstream serglycin effector, and that the capacity of self-renewal in NPC CSCs is possibly maintained by ECM serglycin-activating ERK signaling pathway.

### Serglycin induces CD44 expression to potentiate its self-renewal capacity by activating the MAPK pathway

**Figure 5:**
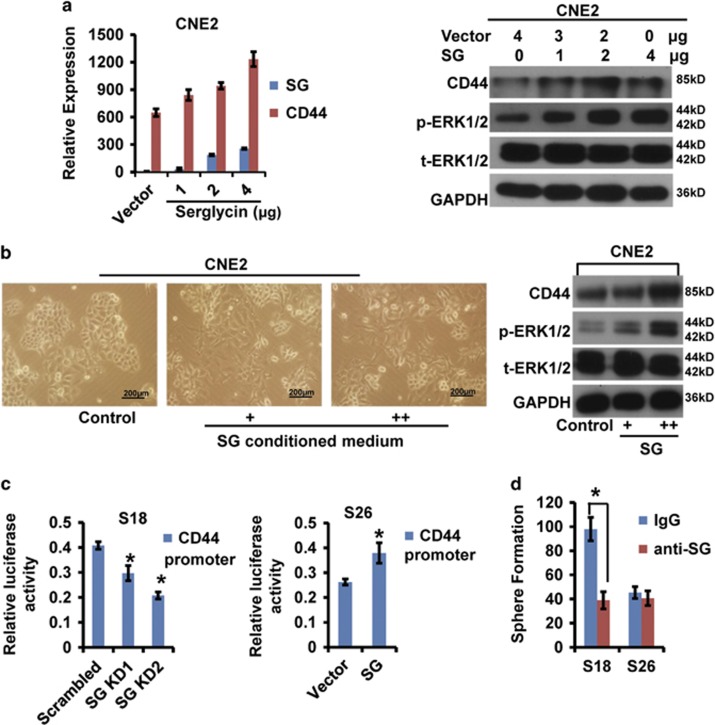
Serglycin-mediated CD44 upregulation by activating the MAPK pathway. (**a**) CNE2 cells were transiently transfected with serglycin. Serglycin and CD44 mRNA levels were detected by quantitative real-time PCR (left panel). Protein levels of CD44, p-ERK1/2, t-ERK1/2 and GAPDH were determined by western blot analysis (right panel). (**b**) CNE2 cells were stimulated by serglycin-CM from S18 cells. The change in CNE2 cellular morphology (× 40, left panel). Protein levels of CD44, p-ERK1/2, t-ERK1/2 and GAPDH were evaluated by western blot analysis (right panel). (**c**) Analysis of CD44 luciferase activity in serglycin knockdown cells (left panel) and serglycin overexpressing cells (right panel). GLuc activities in buffers with a stabilizer. Data represent the average±S.D., *n*=3; *P<0.05 *versus* control cells. (**d**) Number of spheres formed by S18 and S26 cells after treatment with IgG or anti-serglycin antibody. Data represent the average±S.D., *n*=3; *P<0.05 for S26 cells compared with S18 cells

To further confirm that ECM ligand serglycin proteoglycan induces its receptor CD44 expression, we transiently transfected CNE2 cells with serglycin. As expected, both CD44 mRNA and protein levels increased upon serglycin overexpression in a dose-dependent manner (Figure 5a). Furthermore, phospho-ERK1/2 levels also increased gradually. Interestingly, stimulation of CNE2 cells, with serglycin-CM from S18 cells induced a dose responsible spindle-shaped cellular morphology undergoing EMT (Figure 5b, left panel) and significantly increased CD44 and phospho-ERK1/2 protein levels (Figure 5b, right panel). A CD44 promotor luciferase reporter assay revealed decreased activity of CD44 promotor in stable serglycin knockdown S18 cells and increased activity in stable serglycin overexpressing S26 cells (Figure 5c,Supplementary Figure S2A), which suggest that the activity of CD44 promotor was directly regulated by serglycin-activating specific signaling pathway in accord with other results above. Moreover, neutralizing treatment with an anti-serglycin blocking antibody did not affect S26 cell sphere formation but markedly decreased the number of S18 cell spheres, indicating that NPC CSCs produced abundant ECM serglycin proteoglycan to bind its cell surface adherent molecule CD44 receptor and maintained its self-renewal in an autocrine manner (Figure 5d).

### The MAPK/*β*-catenin pathway is activated in S18 cells

**Figure 6:**
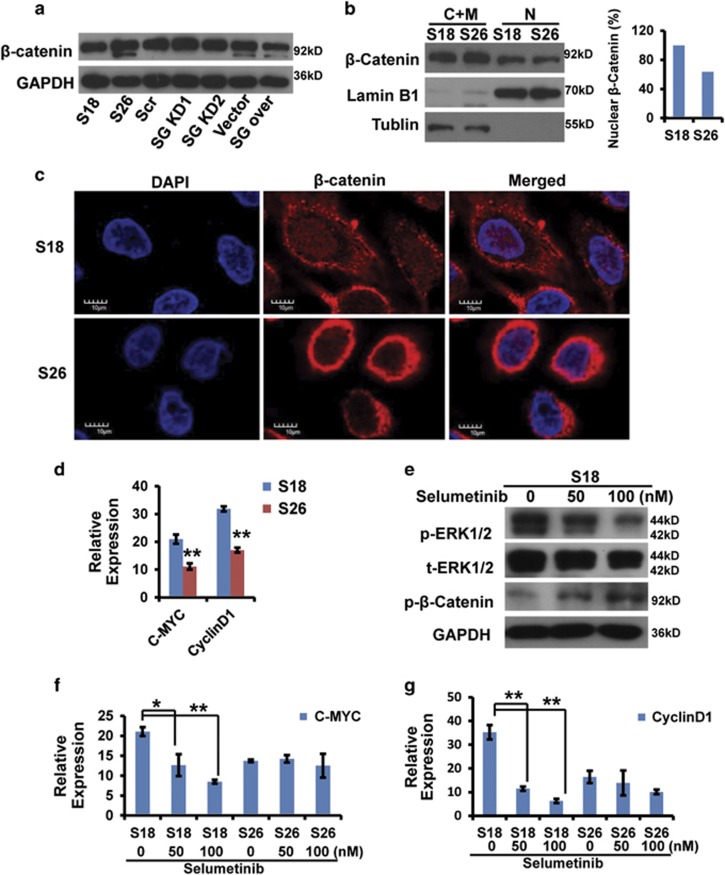
*β*-Catenin localization in S18 and S26 cells. (**a**) *β*-Catenin protein levels in the indicated cells determined by western blot analysis. (**b**) Nuclear (N) and cytosolic/membrane (C+M) fractions of *β*-catenin in S18 and S26 cells determined by western blot analysis. Lamin B1 and tubulin were used as controls for the N and C+M compartment, respectively. (**c**) Localization of *β*-catenin in S18 and S26 cells by confocal immunofluorescence analysis (× 400). (**d**) c-Myc or cyclinD1 mRNA expression (normalized to GAPDH) in S18 and S26 cells. Data represent the average±S.D., *n*=3; **P<0.01 for S26 cells compared with S18 cells. (**e**) Western blot analysis of whole-cell lysates from S18 cells. (**f** and **g**) c-Myc or cyclinD1 mRNA expression (normalized to GAPDH) in S18 and S26 cells treated with selumetinib (ERK inhibitor) for 48 h. Data represent the average±S.D., *n*=3; *P<0.05, **P<0.01 *versus* 0 nM-treated cells

Wnt/*β*-catenin is an important and classical development signaling pathway in embryonic and CSCs and its hyperactivation is involved in the initiation and progression in the majority of malignancies.^32^ CD44 is a common CSC surface marker in subset of cancer and has a key role in their tumorigenesis.^33^ Previous investigations showed that CD44 was an activated *β*-catenin-dependent transcriptional target gene.^34, 35^ Therefore, we next investigated whether serglycin could induce *β*-catenin activation to mediate CD44 transcription. We first evaluated *β*-catenin protein levels in several cell lines (Figure 6a). S26 cells expressed significantly higher levels of *β*-catenin compared with S18 cells. We next investigated *β*-catenin localization in S18 and S26 cells. We examined nuclear (N) and cytosolic/membrane (C+M) fractions and found that more *β*-catenin translocated to the nucleus in S18 cells, whereas it primarily localized to the cell membrane of S26 cells (Figure 6b). We observed similar results by confocal immunofluorescence analysis (Figure 6c). The results above indicated that the *β*-catenin was obviously activated and translocated into the S18 cell nucleus in contrast to S26 cells.

To investigate the upstream signaling to mediate the activation of *β*-catenin, the MAPK-specific inhibitor selumetinib was used, and the results showed the inhibitor effectively suppressed phospho-ERK1/2 expression in S18 cells and then profoundly increased N-terminal phospho-*β*-catenin expression, an inactivated form (Figure 6e). In addition, we examined c-Myc and cyclinD1 mRNA expression, both of them are well-confirmed *β*-catenin-dependent direct target genes.^36^ These two genes were highly expressed in S18 cells compared with S26 cells (Figure 6d). Selumetinib treatment did not affect c-Myc and cyclinD1 expression in S26 cells, but decreased their expression in S18 cells (Figures 6f and g). In addition, IHC results showed that phospho-ERK1/2 was highly expressed in S18 tumor tissues, and *β*-catenin nuclear localization was increased compared with S26 tumor tissues (Supplementary Figure S2B). Therefore, the MAPK/*β*-catenin pathway is highly activated in CSC-like S18 cells in comparison with non-CSC S26 cells.

### Serglycin triggers MAPK/*β*-catenin signaling axis to maintain S18 cell stemness in a positive feedback loop

**Figure 7:**
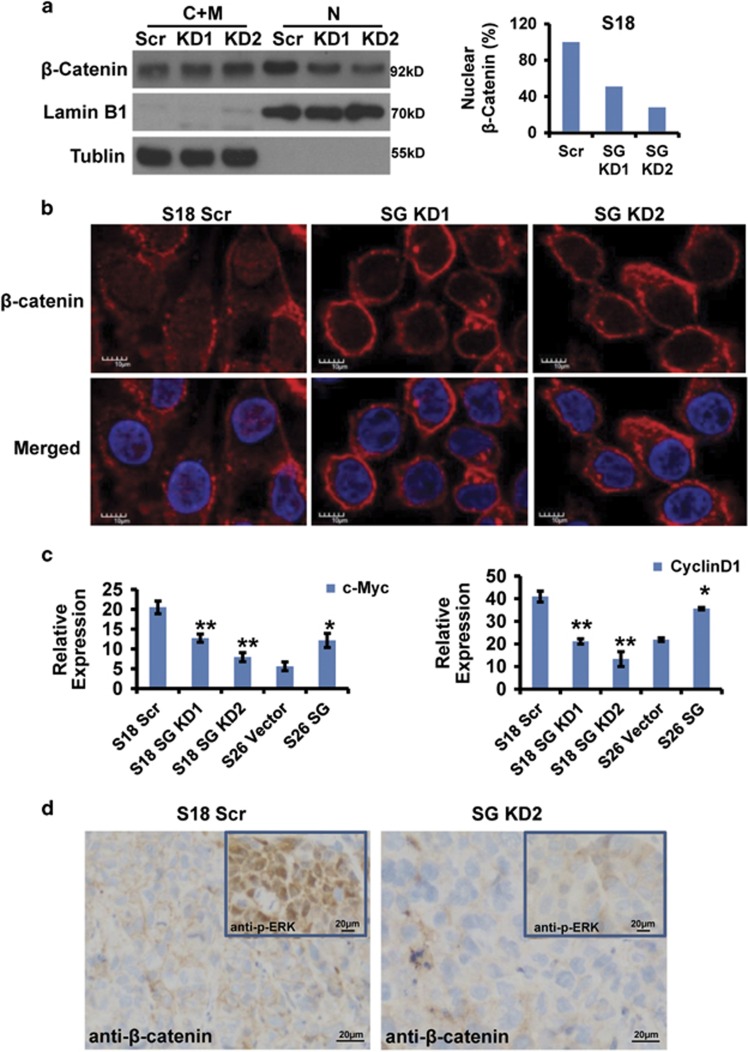
Serglycin induced *β*-catenin activation. (**a**) Nuclear (N) and cytosolic/membrane (C+M) fractions of *β*-catenin in S18 scrambled, S18 SG KD1 and S18 SG KD2 cells determined by western blot analysis. (**b**) Localization of *β*-catenin in S18 scrambled, S18 SG KD1 and S18 SG KD2 cells by confocal immunofluorescence analysis (× 400). (**c**) c-Myc mRNA expression (normalized to GAPDH) in the indicated cell lines. Data represent the average±S.D., *n*=3; *P<0.05, **P<0.01 *versus* DMSO-treated cells. (**d**) IHC staining of *β*-catenin and p-ERK1/2 in S18 scrambled tumor tissues and S18 SG KD2 tumor tissues (200 ×)

ECM, as an essential component of CSC microenvironment, has a key role in maintaining the characteristics of CSCs.^37, 38^ To investigate further the association of ECM serglycin with the activation of *β*-catenin signaling, the fractionation and confocal immunofluorescence analysis of stable serglycin knockdown cells revealed that a fraction of *β*-catenin translocated from the nucleus to the membrane compartment (Figures 7a and b), indicating that the ECM serglycin, as a CD44 ligand, specifically induced nuclear *β*-catenin translocation and triggered *β*-catenin-mediated transcriptional activation of the genes related with stemness. Importantly, we found that c-Myc and cyclinD1 expression changed downstream of *β*-catenin in serglycin knockdown cells or serglycin overexpressing cells (Figure 7c,Supplementary Figure S2D). IHC analysis showed that S18 scrambled tumor tissues highly expressed phospho-ERK1/2, and *β*-catenin localized more strongly to the nucleus compared with S18 SG KD2 tumor tissues (Figure 7d). In summary, autocrine serglycin, as an ECM component and a CD44 ligand, activates *β*-catenin signaling pathway to maintain the self-renewing capacity of NPC CSCs in a positive feedback loop.

## Discussion

CSCs have been suggested to drive tumor initiation and progression and be closely associated with chemoradiotherapy resistance, recurrence and metastasis.^39^ The tumor microenvironment is essential for CSCs self-renewal. ECM is composed of a complex and dynamic array of secreted molecules, such as glycoproteins, proteoglycans, glycosaminoglycans and collagens.^40^ It is a major component of the tumor microenvironment and reciprocally influences the cell's ability to modulate cell growth, survival, motility and differentiation by binding to specific receptors, such as syndecans, integrins and discoidin receptors.^41, 42, 43^ It is clear that genetic modifications in tumor cells initiate and drive malignancy, but tumor-associated ECM is also involved in cancer progression and modulates virtually every tumor cell tumor-associated stromal cell behavior.^44^ Moreover, recent increasing studies showed that the contribution of ECM to maintenance of CSC self-renewal were reported in an autocrine manner in many cancers.^45, 46^ Similarly, this study showed that ECM remodeling pathways were most highly changed between NPC tissues and the non-cancerous tissues by GeneGo Metacore analysis. Moreover, we previously observed that serglycin was the second most highly upregulated gene in S18 cultured cells and S18 xenografts by performing gene expression profiling and high serglycin expression was significantly correlated with EMT and adverse NPC patient outcomes. These studies strongly indicate that serglycin has a critical role in NPC progression and is an independent marker of distant metastases in NPC.^21^

As a typical ECM component, serglycin is a proteoglycan constitutively secreted by highly metastatic NPC cells. Based on Weinberg effect, the EMT generates cells with properties of stem cells.^25, 47^ However, by now no research has been conducted to elucidate the underlying mechanism of serglycin-mediated activation of signaling pathway in CSC-like cells. In this study, we used two single clones (S18 and S26) derived from the representative CNE2 NPC cell line established in our lab,^16, 24^ with highly metastatic S18 cells possessing more cancer stem-like cell characteristics (Figure 1). Other studies have demonstrated that S18 cells have high CSC properties.^48, 49^ Although conventional treatment kills most cancer cells, it is thought to leave CSC behind, allowing for the development of tumor chemoresistance, relapse and metastasis.^50, 51^ As ECM has a crucial role in the establishment and maintenance of stem cell niches,^30, 42^ we explored the role of serglycin in the maintenance of S18 cell stemness. For the first time, we found that serglycin is closely associated with the CSC properties of S18 cells (Figure 2). In addition, serglycin downregulation enhances the sensitivity of S18 cells to chemotherapy (Figure 3), suggesting that serglycin is a promising candidate to target CSCs. However, future research into other genes associated with CSCs remains to be explored.

Cellular surface adherent CD44 molecule is a common CSC marker in several tumors and also a *β*-catenin-mediated transcriptional target.^33, 34, 35^ Our previous studies and other studies have demonstrated that CD44 is a potential NPC CSC marker.^30, 31, 38, 52^ The CD44 receptor primarily binds to hyaluronic acid, but it also serves as a serglycin receptor.^18, 22, 23^ However, so far it is unclear which downstream signaling pathways are activated by the serglycin–CD44 interaction. Exploration of 27 nasopharyngeal samples and 7 NPC cell lines revealed that CD44 and serglycin expression levels were strongly and directly correlated (Figures 4a and b). Importantly, we found that serglycin induced CD44 expression to potentiate the cells' self-renewal capacity by activating the MAPK/*β*-catenin pathway (Figures 4 and 5).The Wnt/*β*-catenin is a crucial CSC signaling pathway and interestingly, NPC CSC-like S18 cells with high-metastic potential expresses nondetectable Wnt (data not shown), which suggests that autocrine ECM serglycin, a CD44 ligand, may be function as Wnt to activate *β*-catenin pathway in NPC CSC-like S18 cells. Remarkably, our recent findings show that Wnt5a is able to activate *β*-catenin-independent signaling pathway to promote stemness characteristics via PKC in the S18 cells.^29^ In other words, both *β*-catenin-independent (non-canonical) and -dependent (canonical) pathways are simultaneously and complementarily activated by autocrine wnt5a and serglycin, respectively, in NPC CSC-like S18 cell line with highly metastic potential.

**Figure 8:**
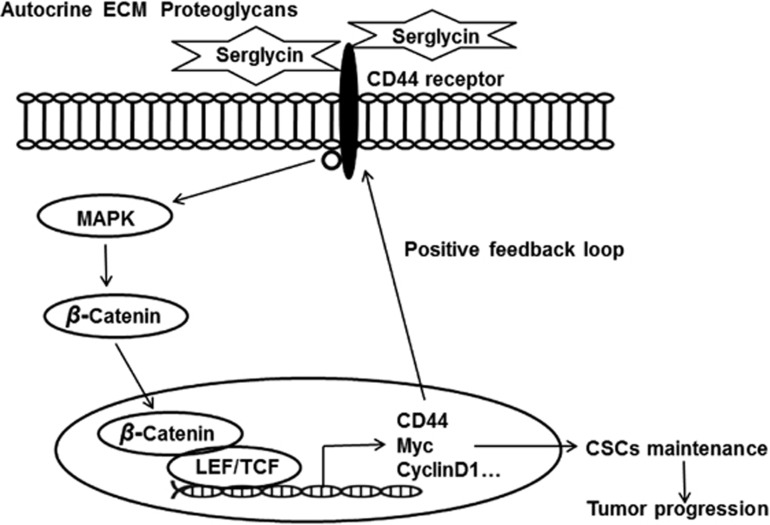
Model of the serglycin–CD44 signaling pathway in NPC. Serglycin binds to CD44 and activates MAPK signaling to subsequently promote *β*-catenin translocation from the membrane compartment to the nucleus. Note the positive feedback loop in which CD44 is the upstream activator and the downstream transcriptional target of the MAPK/*β*-catenin pathway

In conclusion, our findings showed for the first time that ECM serglycin as a CD44 ligand has a crucial role in maintaining NPC cell stemness and contributes to NPC cell metastasis and chemoresistance. In particular, serglycin upregulates its receptor CD44 expression by reciprocally activating the MAPK/*β*-catenin axis. Our novel findings are summarized in Figure 8. These significative findings reveal a fundamental serglycin-dependent positive feedback loop and promote serglycin/CD44 axis and downstream signaling pathway as key CSC targets in NPC.

## Materials and Methods

### Cell culture and tissues

The human NPC cell line CNE2 and its highly metastatic clone S18 and lowly metastatic clone S26 were maintained in Dulbecco modified Eagle medium (DMEM; Gibco, Grand Island, NY, USA) supplemented with 10% FBS (Gibco) in a humidified atmosphere with 5% CO_2_ at 37 °C.^16, 24^ The primary NPC biopsy specimens were collected at the Department for Nasopharyngeal Carcinoma, Sun Yat-Sen University Cancer Center with informed patients' consent and all NPC samples were pathologically confirmed.

### Lentiviral transduction

The BLOCK-iT Lentiviral Pol II miR RNAi system (Invitrogen, Carlsbad, CA, USA) was used to establish S18 stable cell lines. The targets of serglycin shRNA-1 and shRNA-2 were 5′-CTGGTTCTGGAATCCTCAGTT-3′ and 5′-CGCTGCAATCCAGACAGTAAT-3′, respectively. Real-time PCR and immunoblotting were performed to evaluate serglycin knockdown efficiency.

The ViraPower Lentiviral Directional TOPO Expression Kit (Invitrogen) was used to generate the serglycin stable overexpression S26 cell line. Real-time PCR and immunoblotting were performed to evaluate the efficiency of serglycin overexpression.

### Plasmid transfection

Growing CNE2 cells seeded at 1.5 × 10^5^ per well of a six-well tissue culture dish were transiently transfected with different amounts of the serglycin expression plasmid (0, 1, 2 or 4 *μ*g) or the control vector plasmid using X-treme GENE HP DNA transfection reagent (Roche, Mannheim, Germany) according to the manufacturer's instructions.

### Real-time quantitative PCR (qPCR)

Total RNA was extracted from cultured cell lines using TRIzol reagent (Invitrogen) and subjected to reverse transcription using RevertAid First Strand cDNA Synthesis Kit (Thermo, Waltham, MA, USA). Real-time qPCR was performed using a SYBR Green qPCR Kit (kapa) on a CFX96 real-time PCR detection system (Bio-Rad, Hercules, CA, USA). The GAPDH housekeeping gene was used as an internal control to normalize mRNA levels of different genes. Relative changes in expression were calculated using the 2^−ΔΔCt^ (where Ct is the threshold cycle) method. Primer sequences for all genes tested are listed in Supplementary Table S1.

### Detection of serglycin in CM

A total of 1 × 10^6^ cells were plated in 100 mm dishes and cultured in regular medium. After 24 h in culture, cells were starved for 24 h. Culture supernatants were collected, centrifuged at 3000 r.p.m. for 5 min and concentrated with Amicon Ultracentrifuge filters (10 kDa molecular weight cutoff pore size; Millipore, Boston, MA, USA). Serglycin was detected in concentrated CM by immunoblotting.

### Colony formation assay

Cells were counted and plated at 500 cells per well in a six-well plate and incubated for 24 h to allow settling. The cells were treated with a range of cisplatin doses (0, 0.3, 1.2 *μ*M). When the majority of colonies grew to >50 cells, the wells were washed, fixed in methanol for 15 min and stained with crystal violet for 30 min at room temperature. All experiments were independently repeated at least thrice.

### MTS assay

For IC_50_ determination, cells were counted, plated in triplicate at 2000 cells per well (200 *μ*l) in 96-well plates and allowed to grow overnight. For individual groups, cisplatin or 5-fluorouracil was added to the wells in a concentration gradient. Cell viability was measured 48 h later by MTS method (Promega, Madison, WI, USA, G5421). For cell proliferation assays, cells were seeded into 96-well plates at 1000 cells per well and treated with 10% FBS, 0.1% FBS, cisplatin (Sigma, Shanghai, China; 0.8 *μ*M) or 5-fluorouracil (Sigma) (25 *μ*M). Parallel plates were harvested at various times post-seeding. Cell viability was measured by MTS method (Promega, G5421), and the optical density (OD) was measured at 490 nm.

### Immunoblotting

Whole-cell lysates were extracted with RIPA buffer supplemented with 1 : 100 protease inhibitors (Roche). Nuclear or cytoplasmic extracts were prepared with a Nucl-Cyto Preparation Kit (Applygn, Shanghai, China) according to the manufacturer's instructions. The antibodies used for western blotting were as follows: serglycin (Abnova, H00005552-M03, Taibei, China), CD44 (Proteintech, Wuhan, China, 15675-1-AP) and several antibodies from Cell Signaling Technology, Danvers, MA, USA, including cleaved PARP (6525), t-ERK (4780), phospho-ERK1/2 (5726), t-AKT (4691), phospho-AKT (Ser473; 4051), *β*-catenin (9562), and GAPDH (2118). Anti-mouse (W4021) and anti-rabbit (W4011) peroxidase-conjugated secondary antibodies were obtained from Promega. Selumetinib was purchased from Selleck (Shanghai, China) (S1008), U0126 was purchased from Selleck (S1102).

### Sphere formation assay

Cells were seeded in ultra-low attachment six-well plates (Corning, NY, USA) at a density of 1000 cells per well, and cultured in DMEM/F12 medium (Gibco) with 20 ng/ml epidermal growth factor and 20 ng/ml basic fibroblast growth factor and B-27 supplement for approximately 10 days. The spheres were counted under a light microscope. Three independent experiments were performed.

### Immunofluorescence microscopy

Cells were fixed with 4% paraformaldehyde (Tiengene, Guangzhou, China) before being permeabilized in 0.1% Triton X-100, then blocked for 1 h with 5% bovine serum albumin and incubated with primary rabbit *β*-catenin antibody (1:200, Cell Signaling Technology) overnight at 4 °C. Cells were rinsed in PBS and incubated with Alexa Fluor 594-conjugated anti-rabbit IgG (Beyotime, Beijing, China) for 1 h at room temperature. Cells were then stained with DAPI (Invitrogen) before mounting. Images were analyzed using an Olympus immunofluorescence microscope (Tokyo, Japan).

### IHC staining

IHC analysis was performed on three sections. Primary antibodies against *β*-catenin, serglycin and P-ERK were diluted 1 : 100 and were incubated at 4 °C overnight in a humidified container. After washing with PBS three times, tissue slides were treated with a non-biotin horseradish peroxidase detection system according to the manufacturer's instructions (Dako, Glostrup, Denmark).

### Luciferase assay

Cells were plated into 96-well plates and transfected with CD44 luciferase reporter plasmid the next day (GeneCopoeia, Rockville, MD, USA, HPRM10479-PG04). After 2 days, luciferase assays were performed using the Secrete-Pair Dual Luminescence Assay Kit (GeneCopoeia) according to the manufacturer's protocol. The CD44 promoter sequence is listed in Supplementary Figure S3.

### Animal experiments

Athymic nude mice were purchased from Guangdong Medical Laboratory Animal Center (Guangzhou, China) at 4 weeks of age. Tumor cells were suspended in 100 *μ*l PBS containing 50% Matrigel (BD Biosciences, Franklin Lakes, NJ, USA), then injected into the left or right axillary area. The mice were monitored twice per week for palpable tumor formation. All animal experiments were approved by the Institutional Animal Care and Use Committee of the Sun Yat-Sen University Cancer Center.

### Statistical analyses

All statistical analyses were performed using the SPSS (IBM, NY, USA) (version 17) package. Data are represented as the mean±S.D., and a Student's *t*-test (two-tailed) was used to evaluate the significance between any two groups of data. The correlation (*r*) between serglycin and CD44 levels in nasopharyngeal tissues was evaluated using Pearson's correlation analysis (two-tailed). *P*<0.05 was considered statistically significant.

## Abbreviations

NPC: nasopharyngeal carcinoma
CSCs: cancer stem cells
ECM: extracellular matrix
EMT: epithelial-to-mesenchymal transition
MET: mesenchymal-to-epithelial transition
CM: conditioned medium
