# Supplementary material for: Extracellular serglycin upregulates the CD44 receptor in an autocrine manner to maintain self-renewal in nasopharyngeal carcinoma cells by reciprocally activating the MAPK/β-catenin axis
Source: Cell Death Dis. 2016 Nov 3;7(11):e2456–. doi: 10.1038/cddis.2016.287 (PMC5260886; doi:10.1038/cddis.2016.287)

Supplemental Materials

Figure S1

A

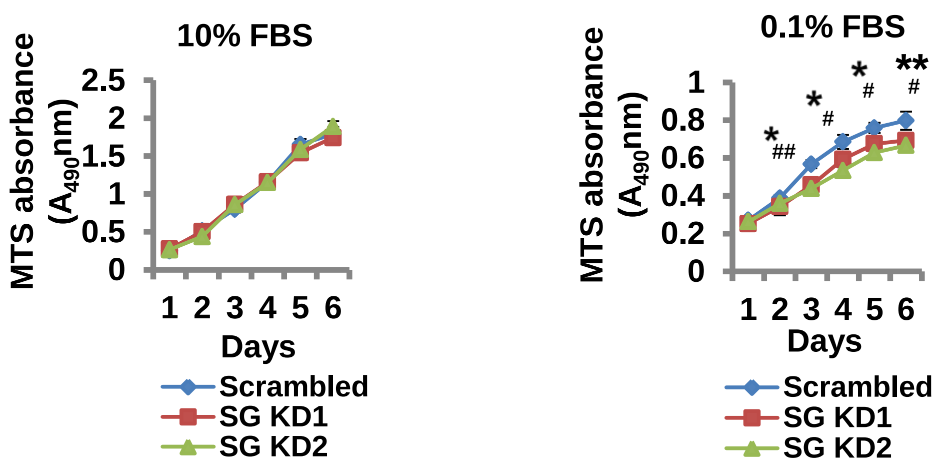

B

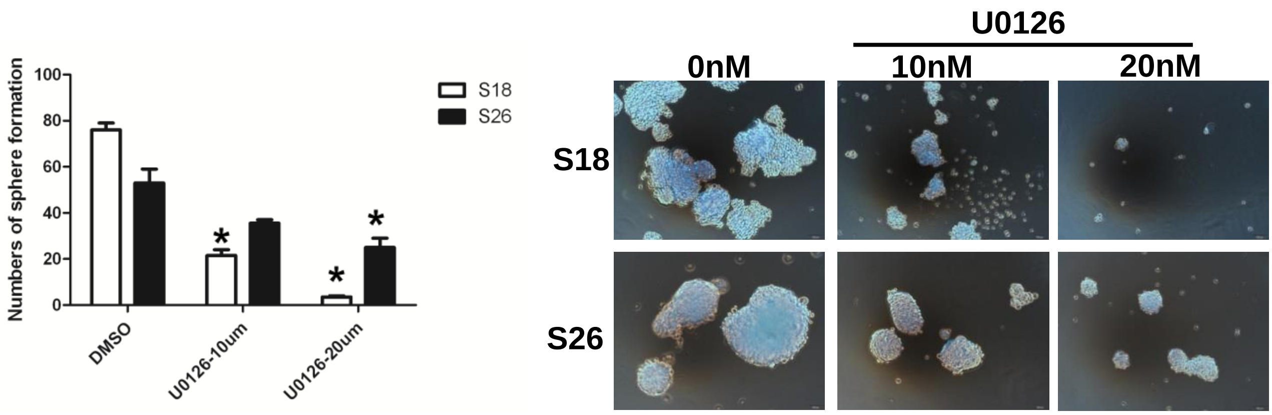

Figure S2

A

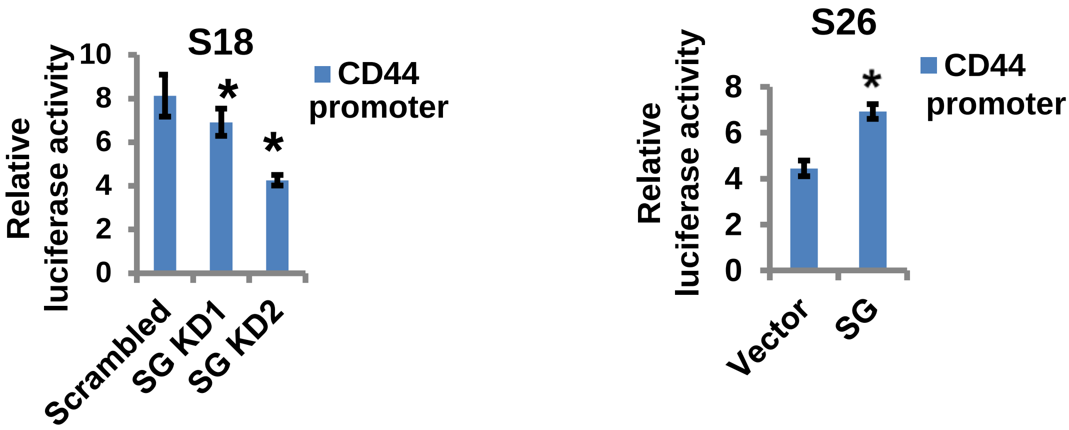
S18
Relative luciferase activity
10
8
6
4
2
0
Scrambled
SG KD1
SG KD2
CD44 promoter
*
*
S26
Relative luciferase activity
8
6
4
2
0
Vector
SG
CD44 promoter
*

B

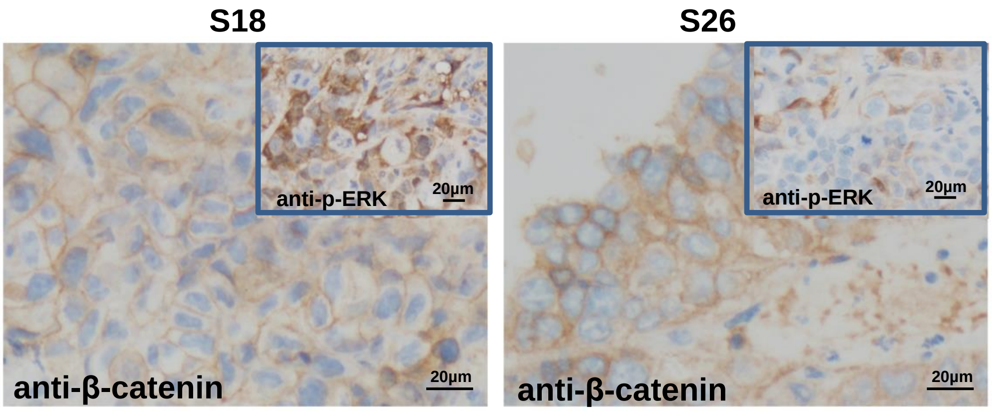
S18
S26
anti-p-ERK
20μm
anti-β-catenin
20μm
anti-p-ERK
20μm
anti-β-catenin
20μm

Table S1

Gene	Forward primer5’-3’	Reverse primer5’-3’
GAPDH	AAGGTCATCCCTGAGCTGAA	TGACAAAGTGGTCGTTGAGG
Oct-4	CTTGCTGCAGAAGTGGGTGGAGGAA	CTGCAGTGTGGGTTTCGGGCA
Bmi1	TGGAGAAGGAATGGTCCACTTC	GTGAGGAAACTGTGGATGAGGA
Nanog	AATACCTCAGCCTCCAGCAGATG	TGCGTCACACCATTGCTATTCTTC
Sox2	AAATGGGAGGGGTGCAAAAGAGGAG	CAGCTGTCATTTGCTGTGGGTGATG
ABCG2	TCATCAGCCTCGATATTCCATCT	GGCCCGTGGAACATAAGTCTT
CD44	TCCATCAAAGGCATTGGGCAG	AACCTGCCGCTTTGCAGGTGT
Serglycin	TATCCTACGCGGAGAGCCAGGTAC	TTCCGTTAGGAAGCCACTCCCAGATC
C-Myc	CGTCTCC ACACATCAGCACAA	TCTTGGCAGCAGGATAGTCCTT
CyclinD1	TATTGCGCTGCTACCGTTGA	CCAATAGCAGCAAACAATGTGAAA

Figure S3

CD44 promoter sequence :

aaataaatactgcgtttgatttccaaacattaaaccatagtatattatagatagatatagagttatcattcaaagtatga
tatttcaatctcaaaaggcttcccctgaagaatattacaaactcttcctctctttaagatctgctgggtaggaaagatgg
gagaaaatgaattaatgtttacacagaaaggaggataatgggggcaaaaataatagatgaacgtatgggtggatgagaga
atggataaaatgataggtggatatgttgatcttggacagatgggaaatgagtggatatatcaataaacagatatgtgggt
ggatgggtggagaagaggatggtggatggttgtggttttatgaagagatgtgaaaaaggaagtgtggaatgatggatgag
aagttgtatgggaagatgaatagaagaataggtggttgaataaattaaaaggtgtgtggttggatgaatgaatgagtggg
atgatagatggacctaagtggttagtggatggacaggaggatggatggatgtgagagccccagaaggacataaggaaaga
tgggtggatagatggatgggcggatggaaggatatttaggaggatgaatgagcatgtgtgtggagagaggtgcccattca
cactggcttgaacacatgggttagctgagccaaatgccagccctatgacaggccatcagtagctttccctgagctgttct
gccaagaagctaaaattcattcaagccatgtggacttgttattgaggggaaaaagaatgagctctccctctttccacttg
gaagattcaccaactccccacccctcactccccactgtgggcacggaggcactgcgccacccagggcaagacctcgccct
ctctccagctcctctcccaggatatccaacatcctgtgaaacccagagatcttgctccagccggattcagagaaatttag
cgggaaaggagaggccaaaggctgaacccaatggtgcaaggttttacggttcggtcatcctctgtcctgacgccgcgggg
ccagcgggagaagaaagccagtgcgtctctgggcgcaggggccagtggggctcggaggcacaggcaccccgcgacactccagg
ttccccgacccacgtccctggcagccccgattatttacagcctcagcagagcacggggcgggggcagaggggcccgc
ccgggagggctgctacttcttaaaacctctgcgggctgcttagtcacagccccccttgcttgggtgtgtccttcgctcgc
tccctccctccgtcttaggtcactgttttcaacctc

Vector Information

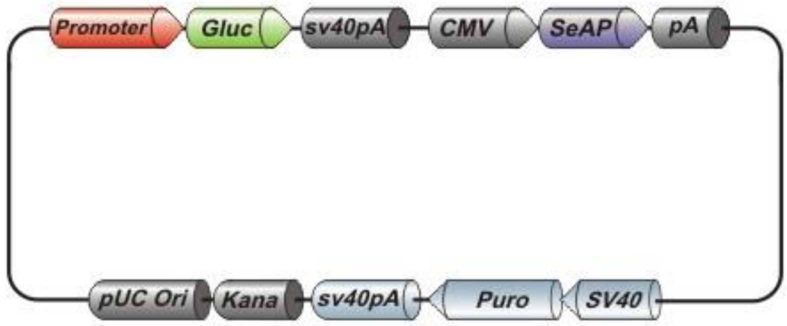

Supplement: Supplemental Material [file cddis2016287x1.pdf]
