# Supplementary material for: Extracellular serglycin upregulates the CD44 receptor in an autocrine manner to maintain self-renewal in nasopharyngeal carcinoma cells by reciprocally activating the MAPK/β-catenin axis
Source: Cell Death Dis. 2016 Nov 3;7(11):e2456–. doi: 10.1038/cddis.2016.287 (PMC5260886; doi:10.1038/cddis.2016.287)
Supplement: Supplemental Table and Figure Legend [file cddis2016287x2.docx]

Figure S1. (A) The cellular growth rate of S18 Scrambled cells, SG KD1 cells and SG KD2 cells in 10% FBS (left panel) or 0.1% FBS (right panel). (B) The effect of U0126 (0 nM, 10 µM, 20 µM) on tumor sphere formation by S18 or S26 cells. The number of spheres is shown in the right panel. Data represent the average±S.D., n=3; **P*< 0.05 vs. 0 nM-treated cells.

Figure S2. (A) Analysis of CD44-luciferase activity in serglycin knockdown cells (left panel) and serglycin overexpressing cells (right panel). GLuc activities in buffers without a stabilizer, Data represent the average±S.D., n=3; **P*<0.05 vs. control cells. (B) The expression of c-Myc and cyclinD1 was detected by quantitative real-time PCR in S18 and S26 cells. Data represent the average±S.D., n=3; ***P*<0.01 vs. S18 cells. (C) The expression of cyclinD1 was detected by quantitative real-time PCR in S18 and S26 cells treated with selumetinib for 48 h, Data represent the average±S.D., n=3; ***P*<0.01 vs. 0 nM-treated cells. (D) The expression of cyclinD1 was detected by quantitative real-time PCR in S18 Scrambled cells, SG KD1 cells, SG KD2 cells, S26 Vector cells and S26 SG cells. Data represent the average±S.D., n=3; **P*<0.05, ***P*<0.01 vs. control cells.

Figure S3. The sequence of CD44 promoter and vector information used in the study.

Table S1. The specific PCR primers used in the study.
